# Who gets connected? Open government data and access to intercity innovation networks

**DOI:** 10.1371/journal.pone.0352359

**Published:** 2026-09-16

**Authors:** Yutong Liu

**Affiliations:** School of Economics and Trade, Hunan University, Changsha, Hunan, China; Bangladesh Rice Research Institute, BANGLADESH

## Abstract

Who gains access to intercity innovation networks when governments publish data online? We examine this question using the staggered launch of municipal open government data (OGD) platforms across Chinese prefecture-level cities from 2010 to 2023. Using city-pair patent records reconstructed as unique unordered pair-year observations, a difference-in-differences (DID) design estimates a 0.169-log-point increase in intercity joint patenting after platform adoption. This effect is not uniform. The increase is larger among cities with fewer pre-treatment partners and weaker commercial-credit, financial, digital, and market-support environments, precisely where private information channels are thinnest. Platform adoption is also associated with broader partner networks, stronger bilateral ties, and more cross-province collaboration. The evidence is consistent with standardized public information substituting for missing private search, reputation, and verification channels. However, it does not establish portal use as the causal mediator or demonstrate a decline in overall spatial inequality. The findings identify a network-entry margin through which public data infrastructure can generate unequal local returns.

## 1. Introduction

Cities do not enter innovation networks on equal informational terms. A specialized manufacturer in a smaller city may possess valuable technology, yet potential research and development (R&D) partners elsewhere may know little about the firm, the local market, or the reliability of the surrounding institutional environment. Before collaboration begins, distant actors must identify a possible match, compare it with alternatives, and decide whether the prospective partner can be trusted. Core cities face a lower informational burden because dense networks generate referrals, repeated interaction, reputational signals, and specialized intermediaries. Initially less-connected cities lack many of these private channels. Their disadvantage is therefore not limited to resources or technological capability; it also concerns whether capability can be discovered and evaluated. Research on the geography of innovation has established that proximity, agglomeration, skilled mobility, prior ties, and the concentration of complex activity shape collaboration networks [1–12]. Less attention has been paid to the public information infrastructure that may alter these entry conditions.

Digital infrastructure may change these conditions, but its distributional consequences are theoretically ambiguous. Lower communication and search costs can broaden access to distant opportunities. At the same time, firms and cities with stronger absorptive capacity, better digital skills, and richer complementary assets may be better positioned to exploit new information [13–18]. A broadband network, an analytical platform, and an open-data portal are therefore unlikely to have identical spatial effects. Technologies that mainly augment computing or analytical capacity may reinforce existing strength. Infrastructure that organizes fragmented public information may have a different effect because it relaxes a constraint that is more binding for unfamiliar or weakly connected places [7,19–23]. The relevant question is not simply whether a digital policy raises innovation on average, but which actors benefit when a specific informational barrier is reduced.

Municipal open government data (OGD) platforms provide a useful setting for studying this question. These portals collect administrative and socioeconomic records that were previously dispersed across departmental websites, yearbooks, notices, and policy documents. The OGD literature has documented effects on transparency, accountability, public participation, entrepreneurship, service delivery, and aggregate economic or innovation outcomes [22–37]. It has also shown that publication alone does not create value. Data must be discoverable, sufficiently current, comparable across sources, and usable by organizations with the capacity to interpret them [23,27–30,34–37]. This literature explains why cities may receive different returns from nominally similar platforms, but it says less about whether public data change the relational structure of innovation. In particular, we know relatively little about whether OGD changes which cities acquire external partners and whether those changes depend on initial network position.

A network-entry perspective changes both the outcome of interest and the distributional question. An increase in total patenting may arise because established innovation centers become more productive without altering access to collaboration. By contrast, an increase in partner breadth or cross-province ties indicates that actors are reaching beyond existing local and regional circuits. We measure this relational margin with intercity joint patent applications, the number of partner cities, the strength of bilateral ties, and the geographic reach of collaboration. Joint patents do not capture every form of knowledge exchange, but they record formal co-production of intellectual property and are more closely tied to intentional collaboration than broad patent counts [2,3,38–45]. These measures allow us to distinguish greater activity within an established core from entry by initially less-connected cities.

The proposed mechanism begins with three frictions in partner formation. Discovery friction limits awareness of potential collaborators. Comparability friction makes it difficult to evaluate alternatives when information is recorded under different definitions or formats. Verification friction arises when claims about local conditions or organizational reliability cannot be checked against a credible source. A municipal portal can reduce these costs by consolidating records, applying common metadata, reporting update dates, and preserving government provenance. Platform launch can also signal administrative modernization or a commitment to transparency. The empirical implications differ. An information channel should be especially relevant to cross-city relationships and to places that lack private search and verification channels, whereas a broad reform or government-quality signal may raise innovation more generally.

The paper develops two competing but compatible predictions. Structural compensation occurs when standardized public information substitutes for information normally supplied by reputation, referrals, repeated interaction, and market intermediaries. It predicts larger marginal gains for cities that begin with fewer partners or weaker verification and matching environments. The policy-capacity view emphasizes complementarities instead. Governments need the capacity to publish reliable data, and users need the skills and organizational resources to recognize and apply them. This perspective predicts larger effects in already capable places. The two mechanisms need not be mutually exclusive. Compensation can operate above a minimum capability threshold, while cumulative advantage may dominate when data quality is poor or productive reuse requires sophisticated analytics and proprietary complements.

China offers a useful empirical setting because municipal platforms were introduced at different times through decentralized local initiatives. There was no single date on which all cities became treated. During the same period, intercity inventive collaboration expanded quickly but remained concentrated in a small set of network hubs. We use the staggered platform launches to estimate a city-year DID model with city and year fixed effects and time-varying controls. Event-time estimates examine pre-treatment dynamics. Balanced-panel and common-sample specifications address sample construction, while alternative transformations, early-adopter exclusions, and policy-group-by-year fixed effects evaluate sensitivity to outcome scaling, treatment timing, and overlapping digital or innovation initiatives.

The patent data originate at the city-pair level. We retain each unordered pair once in each year, assign the pair's collaboration to both endpoint cities, and aggregate the records to city-year outcomes. This construction prevents directional duplicates from inflating joint patent counts or partner breadth. We also form a balanced outcome panel in which city-years without observed intercity collaboration are coded as zero. The resulting measures capture collaboration volume, network breadth, bilateral intensity, and geographic reach on a common basis.

The estimates yield four main findings. First, municipal OGD adoption raises intercity joint patenting by 0.169 log points in the preferred specification. The estimate remains similar when zero-collaboration city-years are added and when the model absorbs annual shocks shared by cities exposed to other digital and innovation policies. Second, platform adoption broadens partner networks, strengthens bilateral ties, and increases cross-province collaboration. Third, the response is much larger among cities with fewer pre-treatment partners. Fourth, the effect declines as baseline commercial-credit, financial, digital, market-support, and technology-contract conditions improve, while highway length does not produce the same moderation pattern. The results therefore point to a network-entry response concentrated where established information and matching channels are relatively weak.

The paper contributes to three literatures. First, it extends research on OGD value creation from average local outcomes to the formation of intercity relationships. This distinction matters because an increase in innovation within established centers and entry by new network participants have different distributional implications. Second, it adds an informational mechanism to the geography of innovation. Existing work emphasizes distance, agglomeration, absorptive capacity, and prior ties; our framework shows how standardized public information can partly substitute for the private information embedded in established networks. Third, it clarifies the meaning of an inclusive digital effect. Larger gains among initially less-connected cities indicate improved access at the network margin, but they do not by themselves show that the national Gini coefficient or the concentration of collaboration has fallen. The contribution is therefore narrower and more precise: public data infrastructure can change who gains access to formal external collaboration even when the aggregate network remains highly unequal.

The remainder of the paper proceeds as follows. Section 2 develops the literature and theoretical framework. Section 3 describes China’s municipal OGD setting, the data construction, and the DID design. Section 4 presents the results. Section 5 discusses theoretical implications, boundary conditions, competing explanations, policy design, and limitations. Section 6 concludes.

## 2. Literature and theoretical framework

### 2.1. OGD value creation, digital divides, and the unresolved network question

OGD research has developed around two broad questions. The first concerns public governance: whether data disclosure improves transparency, accountability, participation, and administrative performance [22–33]. The second concerns economic reuse: whether firms, entrepreneurs, and civil-society organizations can transform public records into market intelligence, products, services, or process improvements [34]. The second question shifts attention from publication to value creation. Government data have no uniform economic effect because users differ in their objectives, organizational routines, technical skills, and access to complementary private data. Reviews of OGD ecosystems accordingly treat value as an outcome of interactions among data providers, intermediaries, users, legal rules, and technical infrastructure [23,36].

Research on reuse identifies several points at which this process can break down. Potential users may not know that a dataset exists, may judge it irrelevant or unreliable, may face restrictive formats, or may lack the staff and software needed to incorporate it into decisions [27–30,35]. Studies of private-sector reuse describe a progression from awareness and perceived business value to organizational commitment and implementation [37]. This perspective explains why platform counts or dataset counts are incomplete measures of policy effectiveness. It also generates a digital-divide prediction: locations with stronger administrative capacity and more sophisticated users may capture disproportionate gains from nominally open information.

These insights have rarely been connected to the formation of innovation networks. Aggregate patenting or firm entry can rise without changing who collaborates with whom. Network formation is distinctive because actors must assess a partner before the joint output exists. Established hubs possess an informational advantage generated by prior ties, referrals, intermediaries, and accumulated reputation [38–41,46–48]. A city outside these circuits may have technically suitable organizations but remain costly to evaluate. The same public information can therefore be redundant for an established hub and valuable for an unfamiliar city. Studying partner acquisition rather than only output levels reveals whether OGD changes access to external knowledge.

### 2.2. Discovery, comparability, and verification frictions

Intercity collaboration can fail before technical compatibility is fully assessed. We distinguish three information frictions. Discovery friction arises when potential partners do not know which firms, laboratories, technologies, or procurement opportunities exist in another city. Comparability friction arises when relevant information exists but uses inconsistent classifications, geographic units, reporting dates, or file formats. Verification friction arises when claims cannot be checked against a source with recognized provenance. These frictions are related to information asymmetry, but they occur at different stages of search. Discovery limits the set of candidates considered; comparability impedes ranking; verification affects the credibility of the final assessment [49,50].

A municipal portal can reduce these costs without supplying a scientific input to the R&D project itself. Centralized search lowers the cost of locating records. Standardized metadata, persistent identifiers, and update histories facilitate comparison across jurisdictions and over time. Government provenance can provide a common reference point for checking local conditions. Data on industrial structure, transport, procurement, land use, market supervision, public services, and administrative procedures may help users assess whether a location offers a viable environment for collaboration. The relevant economic function is not that a particular dataset mechanically produces a patent. It is that organized public information can reduce uncertainty during partner search and evaluation.

Portal-use logs are unavailable, so the empirical analysis evaluates this channel through collaboration patterns and heterogeneity rather than through direct observation of individual searches.

Information can also matter indirectly. A functioning portal may make a local government more legible to outsiders and may signal administrative competence, transparency, or reform commitment. These effects can reinforce direct informational value, but they are conceptually distinct. Direct search and verification effects should be especially visible in relational outcomes and in cities where private information channels are scarce, while a broad quality signal may affect a wider range of local outcomes.

### 2.3. Observability, verification, and signaling

Observability, verification, and signaling describe different changes in the information environment. Observability improves when an attribute that was previously difficult to locate becomes visible. Verification requires more: an outsider must be able to trace the information to a credible source, understand its definition, and judge whether it is current. Signaling does not require the user to inspect a particular record. The act of launching and maintaining a portal may itself communicate government quality or policy orientation. A single platform can perform all three functions, but the functions are not interchangeable. A searchable list of industrial facilities improves observability; consistent definitions and provenance support verification; regular updates and institutional support may generate a signal about administrative capability.

This distinction organizes the empirical tests. If platform launch operates mainly through a general government-quality signal, one might expect broad increases in patenting and similar effects across cities with different network positions. If the central effect is lower search and verification costs, the response should be more visible in cross-city collaboration, partner acquisition, and relationships that cross provincial boundaries. It should also be stronger where prior ties and market intermediaries provide fewer alternative sources of information. These predictions allow competing interpretations to be compared against a coherent set of observable outcomes.

### 2.4. Public information and network formation

A potential tie between organizations in cities i and j forms when expected collaborative surplus exceeds the costs of search, evaluation, verification, coordination, and contracting:


$$\begin{array}{c} {\text{B}}_{\text{ij}}-\tau_{\text{i}}({\text{D}}_{\text{it}},{\text{Z}}_{\text{i}})-\tau_{\text{j}}({\text{D}}_{\text{jt}},{\text{Z}}_{\text{j}})-\chi_{\text{ij}}>\text{0}. \end{array}$$ (1)

Here Bᵢⱼ is the expected surplus from collaboration; τᵢ and τⱼ are city-specific information costs that depend on platform availability D and baseline information conditions Z; and χᵢⱼ contains bilateral barriers such as distance, technological mismatch, and coordination costs. Platform availability can reduce τ by improving observability, comparability, or credibility. It does not create technological complementarity; it changes whether an existing but previously unrecognized match exceeds the formation threshold. Aggregating across potential partners produces two margins. More pairs may form, increasing partner breadth. Existing partners may also undertake more joint work when uncertainty declines, increasing bilateral tie strength. The framework therefore links the information mechanism to both extensive and intensive network outcomes.

### 2.5. Network embeddedness and the marginal value of OGD

Prior network embeddedness supplies private information that can substitute for public disclosure. Repeated ties reveal performance, generate shared routines, and produce referrals to other collaborators. Central network positions also confer reputational benefits because outsiders can infer quality from the identity and number of existing partners [38–41,46–48]. These mechanisms lower information costs even in the absence of a public portal. OGD should therefore have diminishing marginal informational value as private network information becomes abundant.

This argument concerns relational position, not a general label of disadvantage. A geographically central or economically large city may still have few inventive partners, while a smaller city may be well connected through a university, laboratory, or specialized industrial cluster. We define network peripherality using pre-treatment partner breadth and use initially less-connected to describe the relevant group. This distinction prevents the heterogeneity results from being read as evidence about geographic remoteness, income, or administrative rank.

### 2.6. Structural compensation versus policy capacity

Structural compensation refers to the substitution of standardized public information for missing private search, reputation, referral, and verification channels. It is a constrained form of compensation. OGD does not replace laboratories, skilled researchers, finance, or technological compatibility. It can reduce the informational penalty faced by a place that already has a potentially valuable match but lacks the private channels needed to make that match visible and credible.

Policy capacity emphasizes the complementary resources required on both sides of the platform. Governments must collect, update, document, and publish usable records. Firms and research organizations must possess enough digital and organizational capability to interpret them. Structural compensation should dominate when private information is scarce but portal quality and user capacity exceed a minimum threshold. Cumulative advantage should dominate when data are technically complex, interfaces are unreliable, or productive reuse requires specialized personnel and proprietary complements. The relationship may therefore be non-monotonic: the least capable places cannot use the information, moderately capable but weakly connected places gain the most from lower information costs, and highly capable hubs may capture returns from advanced reuse.

### 2.7. Empirical predictions

Proposition 1. Municipal OGD platform adoption increases intercity joint patent collaboration.

Proposition 2. The response appears in partner breadth, bilateral tie strength, and cross-province relationships.

Proposition 3. The marginal response is larger for cities with fewer pre-treatment collaboration partners.

Proposition 4. The marginal response is larger where baseline commercial-credit, financial, digital, and market-support environments are weaker, provided that portal quality and user capacity exceed a minimum threshold.

Proposition 5. A broad measure of physical transport capacity does not reproduce the moderation pattern associated with information and matching conditions.

## 3. Institutional setting, data, and methods

### 3.1. Municipal OGD development in China

China’s municipal OGD system developed through decentralized local launches rather than a single nationwide implementation date. The China Open Data Index and related reports produced by Fudan University’s Digital and Mobile Governance Laboratory record platform establishment and several dimensions of platform development [51]. A recorded launch marks the creation of a centralized municipal portal, not the attainment of mature data governance.

Platform launch and effective data availability differ substantially. Local portals vary in discoverability, metadata quality, download restrictions, application programming interfaces, update frequency, and responsiveness to users. The treatment therefore captures the average effect of establishing a portal across this variation in implementation quality.

China’s wider data-policy environment changed after 2020. The Beijing International Big Data Exchange was established in March 2021, and the Shanghai Data Exchange opened in November 2021 [52,53]. These institutions govern data circulation and transactions rather than unrestricted public download. A national specification issued in 2025 further formalized the authorized operation of public data [54]. These developments are distinct from municipal OGD, but they underscore the need to account for overlapping digital-policy exposure.

We define dataopen_it_ as one from the first recorded year in which city i operates a municipal OGD portal and zero beforehand. Staggered launch timing provides the treatment variation. Because adoption may reflect administrative capacity and reform priorities, the design combines within-city comparisons, national year effects, time-varying controls, event-time estimates, early-cohort exclusions, and policy-group-by-year fixed effects.

**Fig 1:**
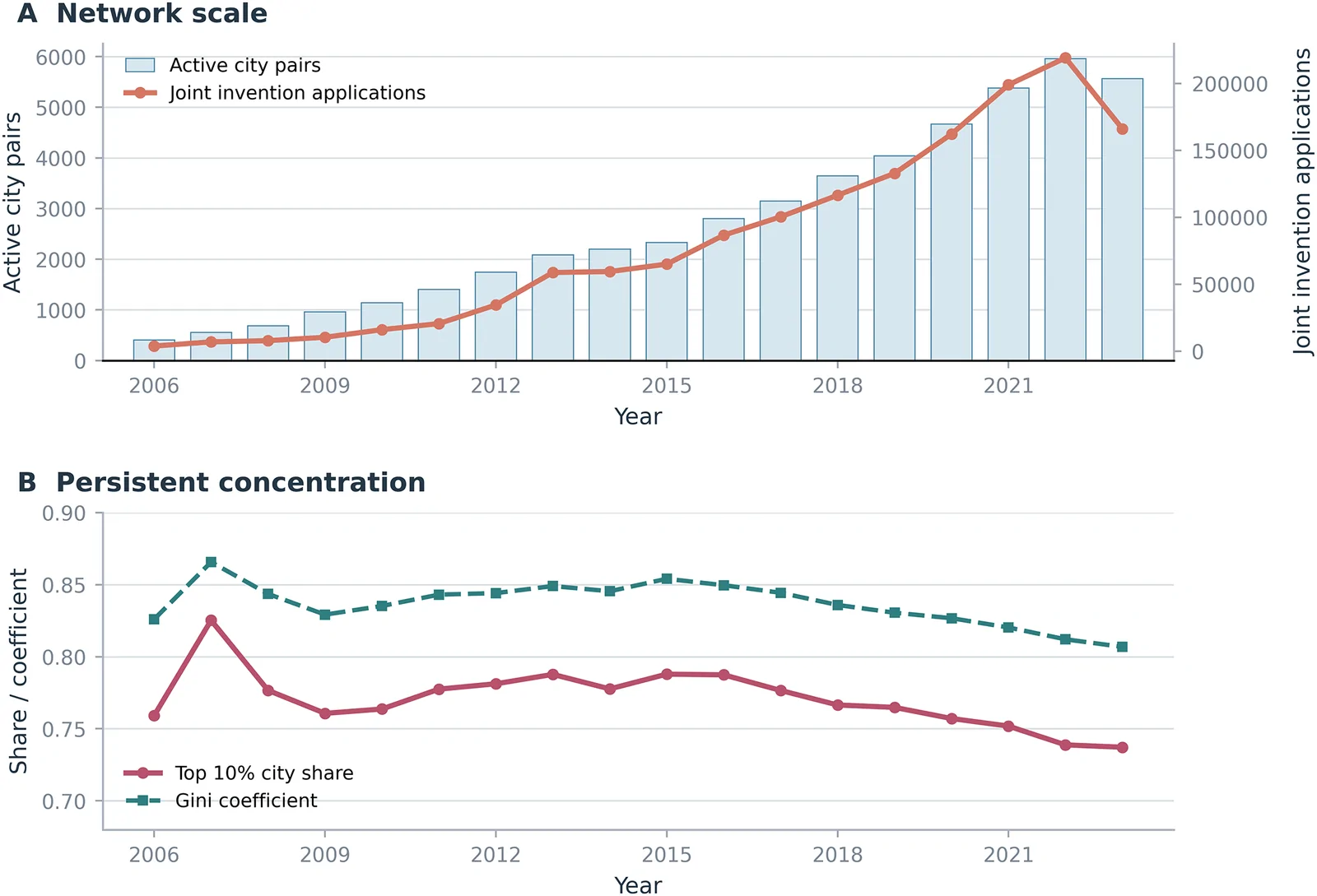
Expansion and concentration of intercity innovation collaboration. Panel A reports annual active city pairs and joint invention applications. Panel B reports the top 10% city share and the city-level Gini coefficient.

Fig 1 shows that intercity collaboration expanded rapidly between 2006 and 2023 while remaining highly concentrated. Active city pairs and joint invention applications increased, but the top-decile city share and the city-level Gini coefficient remained high. This combination of network growth and persistent concentration motivates the analysis of network entry.

**Fig 2:**
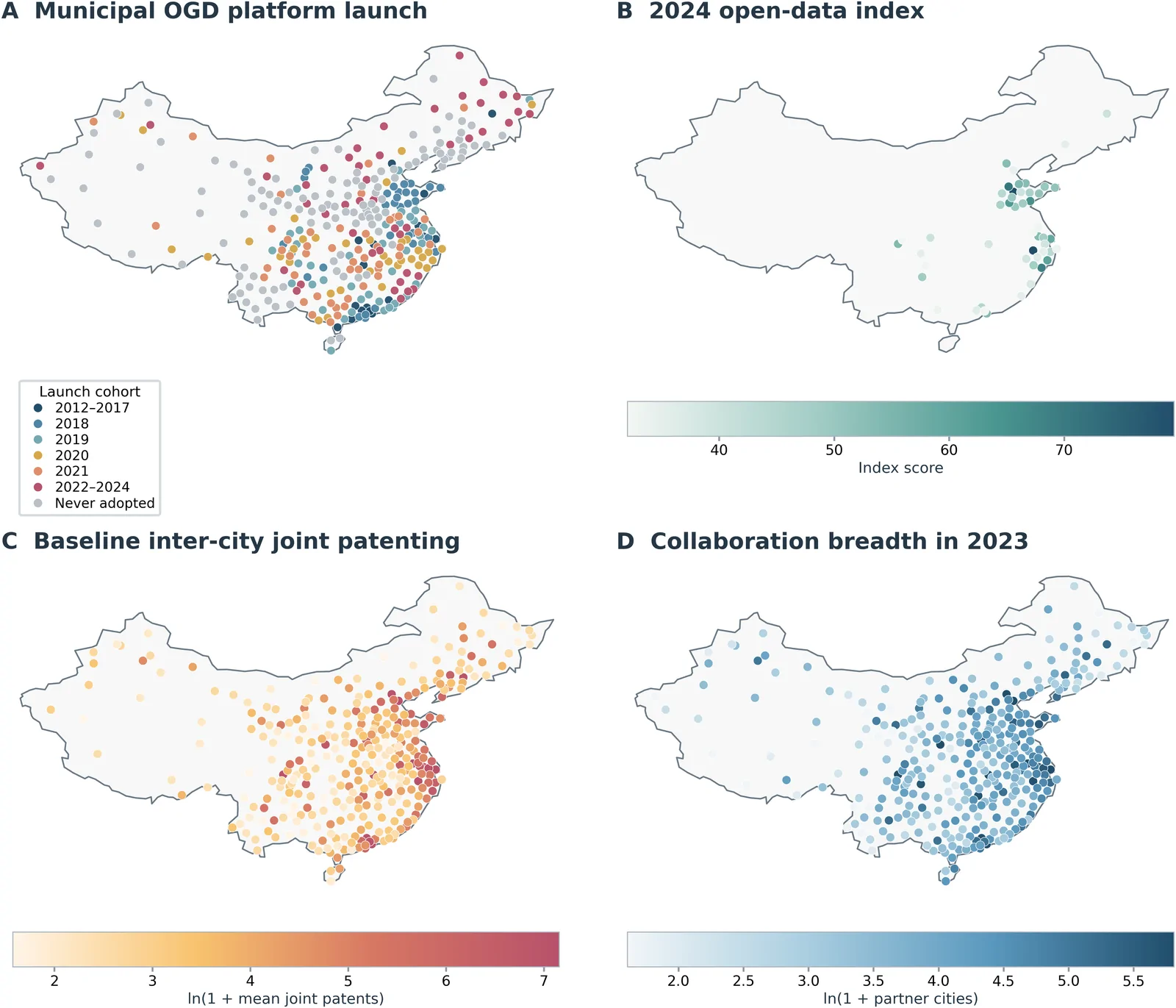
Spatial patterns of public data infrastructure and intercity collaborative innovation. Panels report platform-launch cohorts, the 2024 China Open Data Index, average log intercity joint patenting in 2010–2013, and log partner breadth in 2023. The map uses the public-domain Natural Earth boundary [55].

Fig 2 maps platform-launch cohorts, the 2024 China Open Data Index, baseline collaborative patenting, and 2023 partner breadth. Platform launch and platform quality are related but distinct, and collaborative activity remains geographically concentrated.

### 3.2. Patent collaboration data and outcome construction

Patent records are obtained from the China National Intellectual Property Administration. Applicant addresses are mapped to prefecture-level cities. A patent enters the intercity collaboration measure when its applicants are located in at least two different prefecture-level cities. Coapplications confined to a single city are excluded.

The source represents a relationship between cities i and j in both directions, so the same pair-year appears as i–j and j–i. We standardize city identifiers, sort the two identifiers to form an unordered pair, verify agreement between the directional records, and retain one observation for each city-pair-year. Each pair is then assigned once to both endpoint cities.

City-year joint patenting is the sum of patent counts across unique partner pairs. Partner breadth is the number of distinct partner cities, while maximum and average bilateral strength are calculated across the same unique pair values. Cross-province and within-province outcomes are derived from the provinces of the two endpoint cities. Logarithmic and inverse-hyperbolic-sine transformations are applied only after city-year aggregation.

The dyadic source contains only positive collaboration relationships. Its city-year aggregation therefore yields 4,566 observations for 337 cities. We construct a second panel covering all 337 cities in every year from 2010 to 2023 and code unobserved collaboration as zero, producing 4,718 observations. The economic-control sample contains 4,085 observations for 298 cities, and the preferred full-control sample contains 3,799 observations. Supporting S1 Table reports the inclusion rule for each sample and heterogeneity analysis.

### 3.3. Variables and descriptive statistics

The primary outcome, ln_jointpat, is the natural logarithm of one plus the number of intercity joint patent applications involving a city in a given year. Network outcomes measure partner breadth and the maximum and average number of joint patents across bilateral relationships. Geographic outcomes separate cross-province from within-province collaboration. Broad patent applications and authorizations provide outcome-specificity comparisons.

The control set includes log GDP per capita, log population, tertiary-industry share, urbanization, log R&D expenditure, log government revenue, log number of colleges, and log financial-development index. The marketization measure follows the NERI index [56]. Heterogeneity measures are fixed at their 2011 values, the final year before the first recorded municipal launch. They include partner breadth, the City Commercial Credit Environment Index, financial development, the digital economy, the market-supporting environment, technology-contract activity, and highway length. Fixing these measures before treatment avoids conditioning on responses to platform adoption. Specifications with baseline-outcome trend controls address the possibility that 2011 conditions proxy for persistent city trajectories. Table 1 summarizes the variable definitions, construction, and data sources, while Table 2 reports descriptive statistics for the preferred estimation sample.

**Table 1:** Variable definitions, construction, and source data.

Concept	Variable(s)	Construction and interpretation	Primary source
OGD platform adoption	dataopen	Indicator equal to one from the first recorded municipal portal launch year.	Municipal launch ledger and China Open Data Index reports.
Intercity joint patenting	ln_jointpat	ln(1 + city-year intercity joint patent applications).	CNIPA patent records and geocoded applicant addresses.
Network breadth	ln_partner_cities	ln(1 + distinct partner cities) after unordered pair-year deduplication.	City-pair patent collaboration data.
Bilateral tie strength	ln_max_joint; ln_avg_joint	Log strongest and average joint-patent count across unique partner pairs.	City-pair patent collaboration data.
Geographic reach	ln_crossprov_joint; ln_withinprov_joint	Log joint patents with partners outside/inside the focal province.	City-pair patent collaboration data.
Network position	partners_2011; high_network	Distinct partners in 2011; high network is at or above the 2011 median.	City-pair patent collaboration data.
Commercial credit environment	CEI 2011	Pre-treatment commercial-credit environment used only as a moderator.	City Commercial Credit Environment Index.
Controls and moderators	City characteristics	Economic, fiscal, education, financial, digital, market, technology-contract, and highway measures.	City yearbooks, PKU digital-finance index, NERI index, and related city databases [56].

**Table 2:** Descriptive statistics for the preferred full-control estimation sample.

Variable	Observations	Mean	SD	Min	Max
Intercity joint patents (count)	3,799	371.01	1746.00	1.00	41095.00
ln(1 + intercity joint patents)	3,799	4.11	1.75	0.69	10.62
OGD platform active	3,799	0.22	0.41	0.00	1.00
ln(GDP per capita)	3,799	16.66	0.97	13.62	19.97
ln(population)	3,799	5.84	0.76	3.22	8.07
ln(R&D)	3,799	3.17	0.76	0.00	6.68
ln(government revenue)	3,799	8.20	0.68	5.99	10.62
ln(colleges)	3,799	1.75	0.91	0.69	4.54
ln(financial-development index)	3,799	5.21	0.51	2.97	5.92
Tertiary-industry share (%)	3,799	43.54	10.21	10.15	84.85
Urbanization rate	3,799	0.41	0.22	0.01	1.00

Note: Counts and logarithms are constructed from unique unordered city-pair-year records. The table uses the common 3,799-observation full-control sample.

### 3.4. DID specification

The baseline DID specification is:


$$\begin{array}{c} {\text{Y}}_{\text{it}}={\beta \text{ dataopen}}_{\text{it}}+{\text{X}}_{\text{it}}^{\prime }\gamma +\mu_{\text{i}}+\lambda_{\text{t}}+\varepsilon_{\text{it}}, \end{array}$$ (2)

where Y is a collaboration outcome, X contains time-varying controls, μᵢ denotes city fixed effects, and λ_t_ denotes year fixed effects. Standard errors are clustered by city. Under conditional parallel trends, β captures the average within-city change in the outcome after platform launch. The event-time and sensitivity analyses follow established guidance for staggered-adoption DID designs [57–59].

### 3.5. Event-time and robustness specifications

Event-time coefficients are estimated relative to the year immediately before launch. The robustness analysis uses an inverse-hyperbolic-sine outcome, excludes early adopters, completes unobserved collaboration records with zeros, and compares specifications on a common sample. To absorb differential annual shocks associated with concurrent reforms without assigning unsupported adoption dates, we include policy-group-status-by-year fixed effects. The digital-policy group covers smart-city, Broadband China, national big-data, digital-governance, 5G, and digital-industry initiatives. The innovation-policy group covers comprehensive innovation-reform and national innovation demonstration zones. A broader specification uses membership in any listed policy group.

### 3.6. Heterogeneity and interpretation

Heterogeneity models interact dataopen with pre-treatment moderators. The coefficient on dataopen gives the estimate for the low-baseline group, and the interaction term measures the difference for the high-baseline group. These interactions test whether returns vary with prior network position and information conditions; they are not estimates of a mediated causal pathway.

## 4. Results

### 4.1. Baseline estimates and sample construction

Table 3 separates the consequences of sample construction from those of covariate adjustment. Column 1 uses the 4,566 city-years observed in the positive-collaboration source panel and yields a fixed-effects estimate of 0.269. Column 2 adds the city-years with no observed intercity collaboration, producing a balanced 4,718-observation panel and an estimate of 0.232. Columns 3–5 hold the estimation sample fixed at 3,799 observations, so changes across these columns reflect controls rather than attrition. The coefficient is 0.249 without controls, 0.186 after adding economic controls, and 0.169 with the full control set. The preferred estimate therefore corresponds to an approximate 18.4% increase in intercity joint patenting. Most of the difference between the fixed-effects-only and preferred estimates is attributable to observed city characteristics, not to the treatment of zero outcomes or a changing sample.

**Table 3:** Baseline DID estimates, zero completion, and common-sample comparisons.

Variable	(1) Positive-source FE	(2) Zero-completed FE	(3) Common sample FE	(4) Economic controls	(5) Full controls
dataopen	0.269***(0.053)	0.232***(0.054)	0.249***(0.055)	0.186***(0.054)	0.169***(0.052)
ln_gdp				0.362***(0.137)	0.196(0.150)
ln_pop				1.885**(0.925)	1.919**(0.957)
industry_share3				0.011**(0.005)	0.012**(0.005)
urb_rate				−0.299(0.354)	−0.324(0.345)
ln_RD					−0.004(0.033)
ln_govrevenue					0.278***(0.107)
ln_college					0.187**(0.084)
ln_finance_index					−0.209(0.263)
Observations	4566	4718	3799	3799	3799
Controls	No	No	No	Economic	Full
City FE	Yes	Yes	Yes	Yes	Yes
Year FE	Yes	Yes	Yes	Yes	Yes
Within R-squared	0.013	0.009	0.013	0.034	0.042

Note: All columns include city and year fixed effects. Economic controls are log GDP per capita, log population, tertiary-industry share, and urbanization. Full controls add log R&D expenditure, log government revenue, log colleges, and log financial-development index. Standard errors clustered by city are in parentheses. * p < 0.10, ** p < 0.05, *** p < 0.01.

### 4.2. Dynamic estimates and outcome specificity

**Fig 3:**
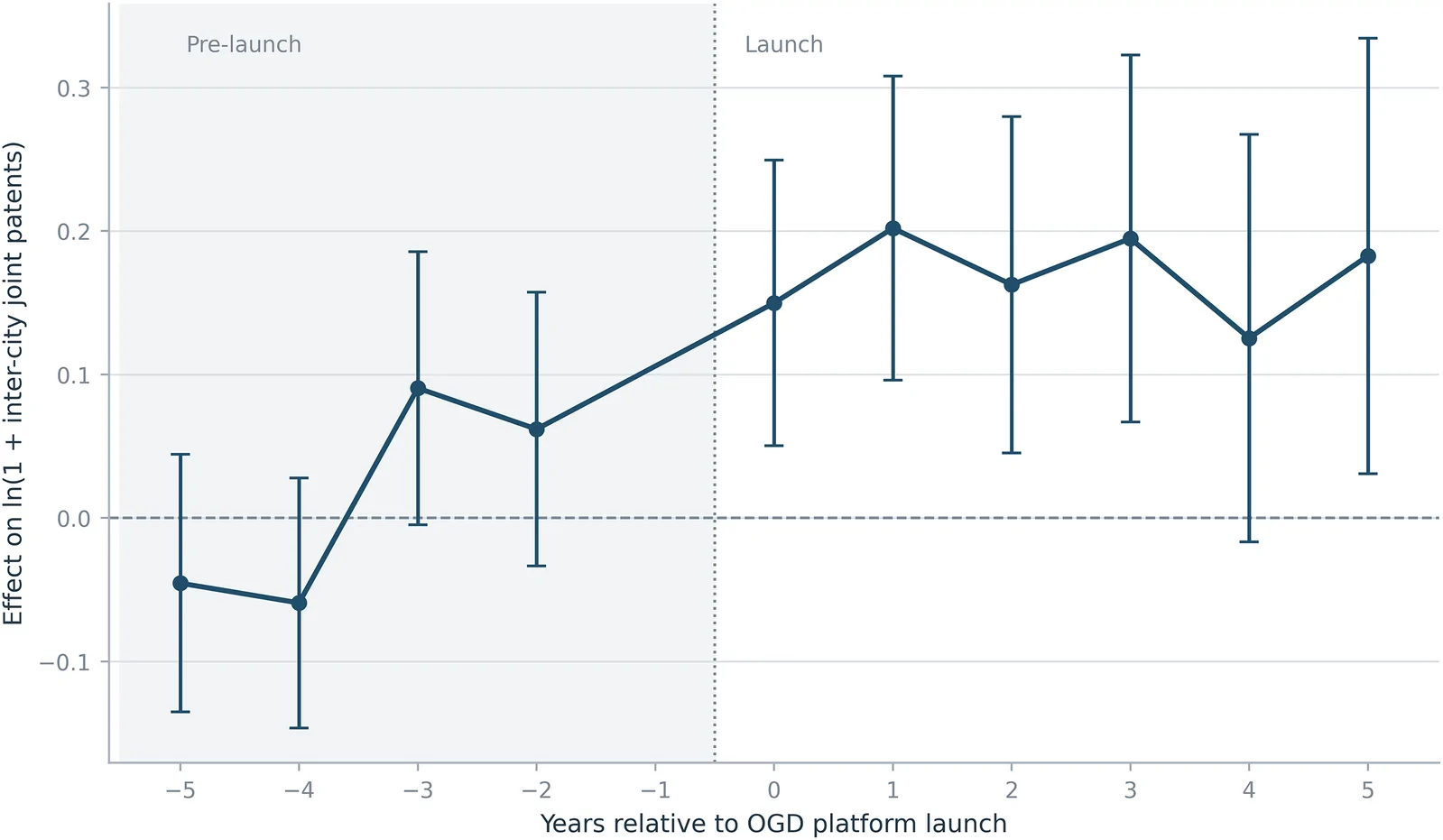
Dynamic DID estimates of OGD adoption and intercity joint patenting. Points are event-time coefficients relative to the year before launch; bars are 95% confidence intervals. Standard errors are clustered by city.

Fig 3 reports the event-time estimates. The pre-launch coefficients are small and statistically indistinguishable from zero at the 5% level, providing no evidence of a systematic rise in collaboration immediately before platform launch. The coefficients turn positive after launch and remain positive in subsequent event years. The dynamic pattern is consistent with a response that begins after the portal becomes available rather than with a smoothly diverging pre-treatment trend.

**Fig 4:**
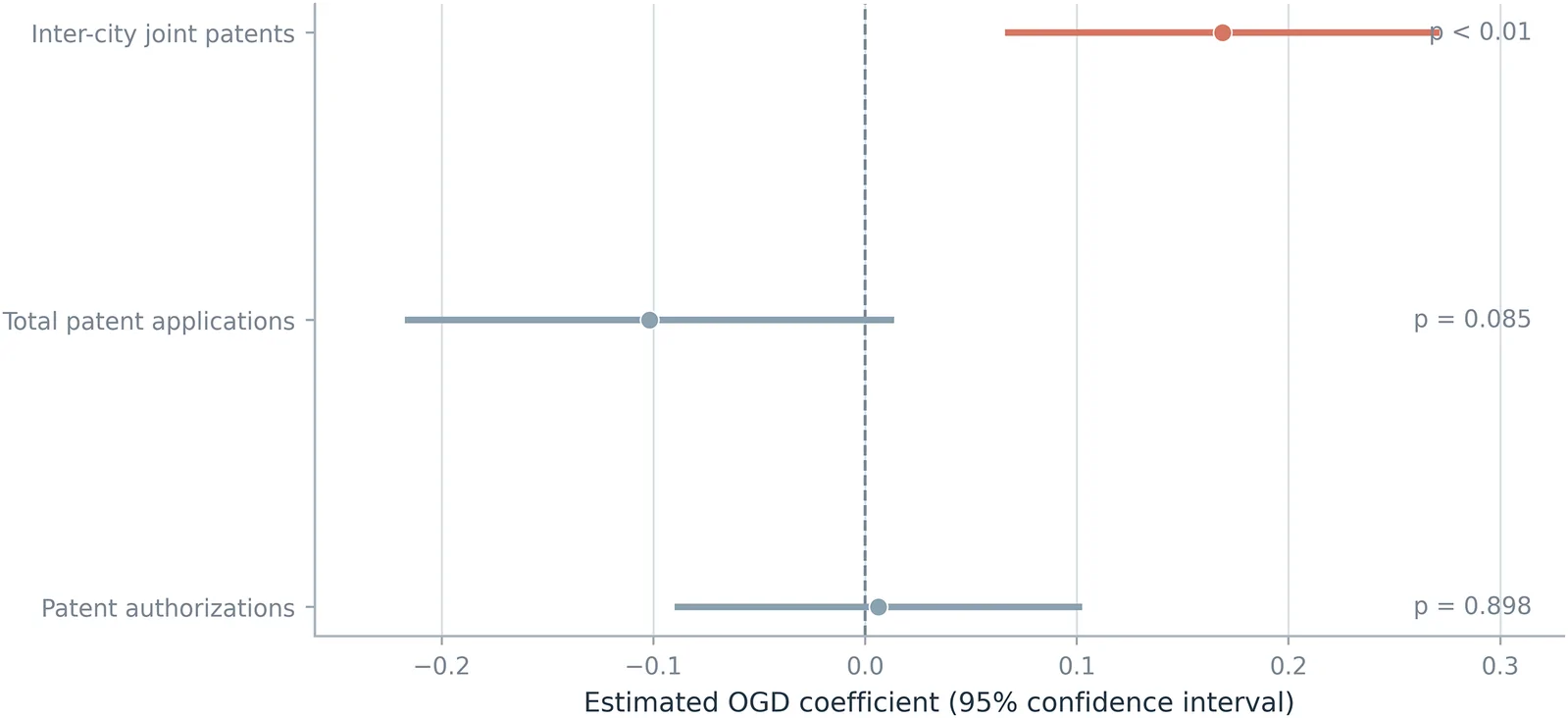
Outcome-specificity checks. Points are OGD coefficients and horizontal bars are 95% confidence intervals for intercity joint patenting and broad local patent outcomes.

Fig 4 compares intercity joint patenting with broad patent applications and authorizations. The estimate is larger and more precise for the collaboration outcome. This outcome specificity matters because a general innovation boom would be expected to raise local patenting more broadly. The observed pattern instead concentrates the response in formal cross-city knowledge production, which is the margin most closely connected to partner search and network entry.

### 4.3. Network structure and sensitivity to concurrent policies

Table 4 shows that platform adoption changes the structure of collaboration as well as its volume. Partner breadth rises by 0.062 log points, maximum bilateral strength by 0.130 log points, and average bilateral strength by 0.093 log points. These estimates correspond to increases of approximately 6.4%, 13.9%, and 9.8%. The simultaneous rise in breadth and tie strength indicates that the main effect is not driven solely by repeated patenting with one incumbent partner. Cities add partners and also deepen at least some bilateral relationships.

**Table 4:** OGD adoption and collaboration-network breadth and bilateral tie strength.

Outcome	OGD coefficient	Approx. effect	Observations	Within R²
Intercity joint patents	0.169***(0.052)	18.4%	3,799	0.042
Partner cities	0.062**(0.030)	6.4%	3,799	0.022
Maximum bilateral strength	0.130**(0.054)	13.9%	3,799	0.044
Average bilateral strength	0.093***(0.034)	9.8%	3,799	0.037

Note: All models include the full control set, city and year fixed effects, and city-clustered standard errors. Standard errors are in parentheses. Approximate percentage effects are calculated as 100 × [exp(β) – 1]. * p < 0.10, ** p < 0.05, *** p < 0.01.

Table 5 reports alternative transformations, cohort restrictions, and overlapping-policy specifications. The estimate is stable under the inverse-hyperbolic-sine transformation and after excluding cities first treated before 2016. It remains between 0.151 and 0.162 when the model absorbs separate annual shocks for digital-policy groups, innovation-policy groups, or membership in any listed policy group.

**Table 5:** Sensitivity checks and overlapping-policy group-by-year fixed effects.

Specification	OGD coefficient	Observations	Within R²
Baseline outcome	0.169***(0.052)	3,799	0.042
Inverse-hyperbolic-sine joint patents	0.171***(0.055)	3,799	0.035
Exclude cities first treated before 2016	0.176***(0.055)	3,682	0.043
Digital-policy-group × year fixed effects	0.160***(0.051)	3,799	0.038
Innovation-policy-group × year fixed effects	0.162***(0.049)	3,799	0.039
Any listed-policy-group × year fixed effects	0.151***(0.049)	3,799	0.035

Note: All specifications include the full control set and city-clustered standard errors. The first three include city and year fixed effects. The final three include city fixed effects and policy-group-status-by-year fixed effects, which absorb national year shocks and differential annual shocks common to cities in the indicated policy groups. Standard errors are in parentheses. * p < 0.10, ** p < 0.05, *** p < 0.01.

### 4.4. Geographic reach and network peripherality

Table 6 separates cross-province from within-province collaboration. Platform adoption raises both outcomes, including a 0.114-log-point increase in cross-province joint patenting. The cross-province result is especially informative because collaboration across provincial borders often involves weaker familiarity and fewer shared institutional routines.

**Table 6:** OGD adoption and the geographic reach of intercity collaboration.

Outcome	OGD coefficient	Observations	Within R²
All intercity joint patents	0.169***(0.052)	3,799	0.042
Cross-province joint patents	0.114**(0.052)	3,762	0.037
Within-province joint patents	0.121**(0.053)	3,762	0.027

Note: All models include the full control set, city and year fixed effects, and city-clustered standard errors. Cross- and within-province outcomes use city-years with classified partner geography, which yields 3,762 observations. Standard errors are in parentheses. * p < 0.10, ** p < 0.05, *** p < 0.01.

Table 7 examines pre-treatment network position. In low-network cities, the OGD coefficient is 0.419 for all intercity joint patents and 0.384 for cross-province joint invention. The interaction with high network position is negative and nearly offsets the low-network estimate, leaving a small and statistically imprecise marginal effect for initially well-connected cities. Adding a high-network linear trend leaves the contrast intact. The pattern is therefore not explained by a simple continuation of different long-run trajectories between high- and low-network cities. It identifies a differential response associated with initial partner breadth.

**Table 7:** OGD adoption by pre-treatment network position.

Variable	(1) All intercity	(2) Cross-province	(3) Cross-province + trend
OGD effect in low-network cities	0.419***(0.077)	0.384***(0.081)	0.351***(0.091)
OGD × high-network position	−0.414***(0.085)	−0.442***(0.093)	−0.383***(0.105)
High-network marginal effect	0.005(0.059)	−0.058(0.058)	−0.032(0.056)
High-network × linear trend			−0.010(0.013)
Observations	3,632	3,595	3,595
Within R²	0.060	0.058	0.058

Note: High network position is defined as a 2011 number of distinct partner cities at or above the sample median. Column 3 additionally includes the interaction between high-network status and a linear time trend. The high-network marginal effect is the sum of the low-network OGD coefficient and the interaction term. All models include the full control set, city and year fixed effects, and city-clustered standard errors. Standard errors are in parentheses. * p < 0.10, ** p < 0.05, *** p < 0.01.

### 4.5. Verification conditions and structural compensation

Table 8 uses the 2011 City Commercial Credit Environment Index (CEI) as a moderator. The OGD estimate is 0.303 in low-CEI cities, and the interaction for high-CEI cities is −0.312. A continuous specification produces the same pattern. Because 2011 is an early pre-treatment baseline, Column 3 interacts baseline collaboration with a linear time trend to absorb persistent trajectories associated with initial network activity. The negative interaction remains statistically significant. The result is therefore not confined to a median split and is not eliminated by allowing cities with different initial collaboration levels to follow different trends. OGD gains remain larger where pre-treatment verification conditions are weaker.

**Table 8:** OGD effects by baseline commercial-credit environment.

Variable	(1) Median split	(2) Continuous CEI	(3) Baseline-trend adjustment
OGD effect in low-CEI cities	0.303***(0.066)	0.142***(0.051)	0.231***(0.068)
OGD × high CEI / standardized CEI	−0.312***(0.076)	−0.136***(0.031)	−0.167**(0.076)
High-CEI marginal effect	−0.010(0.060)		0.064(0.059)
Observations	3,664	3,664	3,530
Within R²	0.048	0.046	0.078

Note: High CEI is defined as a 2011 City Commercial Credit Environment Index above the sample median. In Column 2, CEI is standardized to mean zero and unit standard deviation, so the OGD coefficient is evaluated at mean CEI. Column 3 adds linear trends interacted with baseline collaboration and its square. The high-CEI marginal effect is the sum of the low-CEI OGD coefficient and the interaction term. All models include the full control set, city and year fixed effects, and city-clustered standard errors. Standard errors are in parentheses. * p < 0.10, ** p < 0.05, *** p < 0.01.

Table 9 extends the analysis to financial development, the digital economy, and the market-supporting environment. In every case, the estimate is positive in the low-baseline group and the interaction for the high-baseline group is negative. The high-group marginal effects are small and imprecisely estimated. The common pattern links larger OGD gains to weaker pre-existing information and intermediation environments.

**Table 9:** OGD effects by baseline information and intermediation conditions.

Variable	Financial development	Digital economy	Market-supporting environment
OGD effect in low-baseline group	0.278***(0.072)	0.280***(0.067)	0.274***(0.072)
OGD × high-baseline group	−0.193**(0.083)	−0.270***(0.080)	−0.209**(0.082)
High-baseline marginal effect	0.085(0.063)	0.011(0.063)	0.065(0.063)
Observations	3,629	3,569	3,632
Within R²	0.048	0.048	0.049

Note: Each high-baseline group is defined using values above the 2011 sample median of the indicated moderator. The high-baseline marginal effect is the sum of the low-baseline OGD coefficient and the interaction term. All models include the full control set, city and year fixed effects, and city-clustered standard errors. Standard errors are in parentheses. * p < 0.10, ** p < 0.05, *** p < 0.01.

Table 10 compares technology-contract activity with highway length. The interaction for high technology-contract activity is negative, while the highway interaction is small and statistically insignificant. Established technology markets appear to reduce the marginal value of OGD by supplying alternative matching and verification channels. Highway capacity, which primarily supports physical mobility and face-to-face contact, does not generate the same moderation pattern. This contrast points to a mechanism tied more closely to institutionalized information and matching than to general connectivity.

**Table 10:** Technology-contract market and highway-length moderation.

Variable	(1) Technology-contract market	(2) Highway length
OGD effect in low-baseline group	0.286***(0.085)	0.176**(0.069)
OGD × high-baseline group	−0.204**(0.092)	−0.038(0.076)
High-baseline marginal effect	0.082(0.057)	0.138**(0.060)
Observations	3,632	3,632
Within R²	0.049	0.046

Note: High-baseline groups are defined using values above the 2011 sample medians of technology-contract activity and highway length, respectively. The high-baseline marginal effect is the sum of the low-baseline OGD coefficient and the interaction term. All models include the full control set, city and year fixed effects, and city-clustered standard errors. Standard errors are in parentheses. * p < 0.10, ** p < 0.05, *** p < 0.01.

Across the heterogeneity analyses, OGD gains are largest where prior network, verification, and intermediation channels are weakest. The consistency of this pattern across distinct moderators supports a bounded structural-compensation interpretation.

## 5. Discussion

### 5.1. The theoretical meaning of structural compensation

The central result is a differential network-entry response. Platform adoption produces larger gains in cities that begin with fewer collaboration partners and weaker verification environments. Structural compensation describes this pattern as a form of resource substitution: standardized public information partly replaces information that established networks otherwise supply through reputation, referrals, repeated interaction, and specialized intermediaries.

This interpretation places network embeddedness at the center of OGD value creation. Dense networks transmit knowledge, but they also reveal reliability and reduce uncertainty. Public information adds less when these private informational assets are already abundant. For an initially less-connected city, a portal can lower the penalty of unfamiliarity and bring latent technological complementarities within reach, even without changing the city’s underlying stock of laboratories, researchers, or firms.

The argument also extends the institutional-voids perspective [60]. In emerging markets, organizations and intermediaries often perform matching, information, and enforcement functions that specialized institutions would otherwise provide. OGD can serve as a quasi-public substitute for missing private information infrastructure by standardizing access, provenance, and comparison. Its value may decline as cities accumulate repeated ties, referrals, and reputational capital. Structural compensation is therefore dynamic: it is strongest during network entry and weakens as relational information becomes available through the network itself.

### 5.2. Relation to OGD, digital-divide, and innovation-geography research

The findings reinforce the OGD literature’s emphasis on use rather than publication alone [27–30,34–37,61–64]. The relevant policy asset is not the number of datasets but the capacity of public information to become discoverable, comparable, credible, and useful. This study adds a relational dimension to that argument. OGD value can appear through new external ties, not only through administrative performance or aggregate local innovation.

Structural compensation and absorptive capacity address different parts of the same process. Absorptive capacity determines whether organizations can recognize, assimilate, and apply external information [65]. Structural compensation determines where a reduction in discovery and verification costs has the greatest marginal value. Initially less-connected cities may gain more once they cross a minimum capability threshold, while cities below that threshold remain unable to use even well-organized data. At high levels of capacity, sophisticated reuse may again favor advantaged locations. The two perspectives therefore describe different segments of the capability distribution rather than competing universal laws.

For innovation geography, public information infrastructure complements rather than replaces proximity, agglomeration, and prior network position. Distance still entails legal, technological, and organizational costs. OGD addresses a narrower barrier by making unfamiliar places easier to discover and evaluate. The cross-province results show that reducing this informational component can matter even when other spatial frictions remain.

### 5.3. Boundary conditions: compensation versus cumulative advantage

Structural compensation should be strongest under four conditions: potential collaborators face meaningful uncertainty, private information channels are thin, the portal supplies credible and current information, and users possess enough capability to interpret it. These conditions define an intermediate range in which information is a binding constraint but underlying collaborative capacity already exists.

Cumulative advantage dominates when effective reuse depends on sophisticated analytics, specialized personnel, application programming interfaces, or proprietary complementary data. High-capacity governments may maintain more reliable portals, and high-capacity firms may integrate public information more effectively into search and decision systems. The same policy can therefore compensate for weak private information in one setting and reinforce existing advantage in another.

### 5.4. Competing explanations and evidence interpretation

Platform launch may affect collaboration through channels beyond dataset access. It can signal a government’s commitment to transparency and modernization, coincide with e-government reform or innovation support, and reflect broader improvements in the local business environment. The policy-group-by-year specifications absorb annual shocks shared by cities exposed to major digital and innovation initiatives, reducing the scope for these common policy explanations.

Three features favor the information-friction account over a uniform local innovation shock. The response is concentrated in cross-city collaboration, appears in partner breadth and bilateral ties, and is largest where existing network and verification channels are weak. Highway length does not reproduce this moderation pattern. Physical transport remains important for face-to-face interaction, but the contrast suggests that the observed heterogeneity is more closely related to institutionalized information and matching channels than to general connectivity.

### 5.5. What the results say about spatial inequality

The heterogeneity results concern network peripherality, measured by pre-treatment partner breadth. They do not equate weak network position with geographic remoteness, low income, or administrative rank. Large proportional gains among initially less-connected cities may also have little effect on aggregate concentration because network-core cities account for a dominant share of total collaboration. The results therefore indicate broader access at the margin without implying rapid convergence in the overall network distribution.

### 5.6. Policy implications

A portal intended to support external matching should prioritize usability over nominal dataset counts. Useful features include standardized metadata, persistent identifiers for organizations and jurisdictions, machine-readable formats, stable application programming interfaces, update timestamps, version histories, clear provenance, and accessible correction or verification channels. Interoperability is especially important because external partners compare locations rather than evaluate a city in isolation.

Policy design should distinguish open portals from data exchanges and authorized-operation arrangements. Open portals support broad access and reuse; exchanges and authorized operation govern controlled circulation and products derived from public data [52–54]. These institutions can complement one another, but they create different forms of access and serve different user groups. Evaluation should track availability, reliability, reuse, and interoperability in addition to the date of launch.

Usage analytics would strengthen both management and evaluation. With appropriate privacy safeguards, portal operators could track failed searches, update delays, downloads, application programming interface requests, and unresolved data needs. These records would reveal where users encounter friction and which information products support external matching.

### 5.7. Limitations and future research

First, the institutional setting combines decentralized municipal implementation with strong administrative involvement. Effects may differ in national portals, federal systems, or jurisdictions governed by other legal rules for public information.

Second, city-specific reforms may coincide with platform launch. The policy-group-by-year specifications absorb broad overlapping shocks but cannot capture every local change.

Third, joint patents measure formalized inventive collaboration and omit informal knowledge exchange, contract research without joint ownership, publication networks, supply-chain learning, and unsuccessful searches. They are therefore a lower-bound measure of observable formal collaboration. Their advantage is that they record intentional cross-organizational knowledge production more directly than aggregate patent counts [2,3,38–45].

Fourth, network entry does not guarantee durable or high-quality collaboration. Future research should examine tie survival, repeated collaboration, patent quality, commercialization, and movement toward more central network positions.

Fifth, the data record platform establishment but not the identities, searches, downloads, or datasets used by prospective partners. Linking usage records to subsequent ties would permit a direct test of the proposed informational channel.

Sixth, platform launch does not measure implementation quality. Metadata, updating practices, interfaces, reliability, and actual data availability vary across cities and remain part of the average treatment effect.

## 6. Conclusions

Municipal OGD platforms expand participation in intercity patent collaboration networks. The preferred estimate is 0.169 log points (SE = 0.052) and remains stable across balanced-panel, common-sample, alternative-transformation, cohort-restriction, and overlapping-policy specifications. Platform adoption also broadens partner networks, strengthens bilateral ties, and increases cross-province collaboration.

The gains are largest among cities with fewer pre-treatment partners and weaker verification and intermediation environments. This pattern supports structural compensation: standardized public information is most valuable where private search, reputation, and matching channels are scarce. OGD therefore affects not only how much collaboration occurs, but also which cities gain access to external innovation networks.

For policy, the result shifts attention from portal launch to information usability. Metadata, identifiers, timely updates, interoperability, provenance, and evidence of actual reuse are central to whether public data can support external matching.

## Supporting information

S1 Fig OGD coefficients across collaboration-network outcomes. Points are coefficients and horizontal bars are 95% confidence intervals.
(TIFF)

S2 Fig Marginal OGD effects by baseline commercial-credit environment. Points show low- and high-CEI marginal effects with 95% confidence intervals.
(TIFF)

S1 Table Sample construction and moderator-specific analytic samples. The table reports city and observation counts and the exact inclusion requirement for each analysis.
(DOCX)

S1 Data Workbook containing the city-year analytic panel constructed from unique unordered city-pair-year records and the zero-completed panel for 337 cities from 2010 to 2023. (XLSX)

S2 Data English-language 2011 baseline-moderator file. (CSV)

S3 Data Annual inputs for Fig 1. (CSV)

S4 Data City-level inputs for Fig 2. (CSV)

S5 Data Policy-pilot city lists used for group-by-year robustness specifications. (CSV)

S6 Code Self-contained reproducibility package with scripts, locked requirements, outputs, a data dictionary, source-access documentation, and aggregation documentation. (ZIP)

## References

[1] Jaffe AB, Trajtenberg M, Henderson R. Geographic localization of knowledge spillovers as evidenced by patent citations. Q J Econ. 1993;108(3):577–98.

[2] Breschi S, Lissoni F. Mobility of skilled workers and co-invention networks: an anatomy of localized knowledge flows. J Econ Geogr. 2009;9(4):439–68.

[3] Crescenzi R, Nathan M, Rodríguez-Pose A. Do inventors talk to strangers? On proximity and collaborative knowledge creation. Research Policy. 2016;45(1):177–94. doi: 10.1016/j.respol.2015.07.003

[4] Balland P-A, Jara-Figueroa C, Petralia SG, Steijn MPA, Rigby DL, Hidalgo CA. Complex economic activities concentrate in large cities. Nat Hum Behav. 2020;4(3):248–54. doi: 10.1038/s41562-019-0803-3 31932688

[5] Audretsch DB, Feldman MP. R&D spillovers and the geography of innovation and production. Am Econ Rev. 1996;86(3):630–40.

[6] Boschma RA. Proximity and innovation: a critical assessment. Reg Stud. 2005;39(1):61–74.

[7] Gurstein MB. Open data: empowering the empowered or effective data use for everyone? First Monday. 2011;16(2). doi: 10.5210/fm.v16i2.3316

[8] Porter ME. Clusters and the new economics of competition. Harv Bus Rev. 1998;76(6):77–90. 10187248

[9] Feldman MP, Audretsch DB. Innovation in cities: science-based diversity, specialization and localized competition. Eur Econ Rev. 1999;43(2):409–29.

[10] Carlino GA, Chatterjee S, Hunt RM. Urban density and the rate of invention. Journal of Urban Economics. 2007;61(3):389–419. doi: 10.1016/j.jue.2006.08.003

[11] Bettencourt LMA, Lobo J, Helbing D, Kühnert C, West GB. Growth, innovation, scaling, and the pace of life in cities. Proc Natl Acad Sci U S A. 2007;104(17):7301–6. doi: 10.1073/pnas.0610172104 17438298 PMC1852329

[12] Hidalgo CA, Klinger B, Barabási A-L, Hausmann R. The product space conditions the development of nations. Science. 2007;317(5837):482–7. doi: 10.1126/science.1144581 17656717

[13] Forman C, Goldfarb A, Greenstein S. The internet and local wages: a puzzle. Am Econ Rev. 2012;102(1):556–75.

[14] Goldfarb A, Tucker C. Digital economics. J Econ Lit. 2019;57(1):3–43.

[15] Brynjolfsson E, Rock D, Syverson C. The productivity J-curve: how intangibles complement general purpose technologies. Am Econ J Macroecon. 2021;13(1):333–72.

[16] Farboodi M, Veldkamp L. A growth model of the data economy. Cambridge (MA): National Bureau of Economic Research. 2021.

[17] Nambisan S, Lyytinen K, Majchrzak A, Song M. Digital Innovation Management: Reinventing Innovation Management Research in a Digital World. MIS Quarterly. 2017;41(1):223–38. doi: 10.25300/misq/2017/41:1.03

[18] Graham M, De Sabbata S, Zook MA. Towards a study of information geographies: (im)mutable augmentations and a mapping of the geographies of information. Geography and Environment. 2015;2(1):88–105. doi: 10.1002/geo2.8

[19] Agrawal A, Catalini C, Goldfarb A, Luo H. Slack time and innovation. Organ Sci. 2018;29(6):1056–73.

[20] Bakos JY. Reducing buyer search costs: implications for electronic marketplaces. Manag Sci. 1997;43(12):1676–92.

[21] Shapiro C, Varian HR. Information rules: a strategic guide to the network economy. Boston: Harvard Business School Press. 1999.

[22] Dawes SS. Stewardship and usefulness: policy principles for information-based transparency. Gov Inf Q. 2010;27(4):377–83.

[23] Ubaldi B. Open government data: towards empirical analysis of open government data initiatives. Paris: OECD Publishing. 2013.

[24] Janssen M, Charalabidis Y, Zuiderwijk A. Benefits, adoption barriers and myths of open data and open government. Inf Syst Manag. 2012;29(4):258–68.

[25] Zuiderwijk A, Janssen M. Open data policies, their implementation and impact: a framework for comparison. Gov Inf Q. 2014;31(1):17–29.

[26] Mergel I, Kleibrink A, Sorvik J. Open data outcomes: U.S. cities between product and process innovation. Gov Inf Q. 2018;35(4):622–32.

[27] Safarov I, Meijer A, Grimmelikhuijsen S. Utilization of open government data: A systematic literature review of types, conditions, effects and users. Information Polity. 2017;22(1):1–24. doi: 10.3233/ip-160012

[28] Reggi L, Dawes S. Open government data ecosystems: linking transparency for innovation with transparency for participation and accountability. Gov Inf Q. 2016;33(1):13–23.

[29] Kitchin R. Big Data, new epistemologies and paradigm shifts. Big Data & Society. 2014;1(1). doi: 10.1177/2053951714528481

[30] Jetzek T, Avital M, Bjørn-Andersen N. Generating value from open government data. In: Baskerville R, Chau M, editors. In: Proceedings of the 34th International Conference on Information Systems (ICIS 2013), Milan, Italy. Atlanta (GA): Association for Information Systems; 2013. 1737–56.

[31] Ruijer E, Grimmelikhuijsen S, Meijer A. Open data for democracy: developing a theoretical framework for open data use. Gov Inf Q. 2017;34(1):45–52.

[32] Bertot JC, Jaeger PT, Grimes JM. Using ICTs to create a culture of transparency: e-government and social media as openness and anti-corruption tools for societies. Gov Inf Q. 2010;27(3):264–71.

[33] Attard J, Orlandi F, Scerri S, Auer S. A systematic review of open government data initiatives. Gov Inf Q. 2015;32(4):399–418.

[34] Magalhaes G, Roseira C. Open government data and the private sector: an empirical view on business models and value creation. Gov Inf Q. 2020;37(3):101248. doi: 10.1016/j.giq.2017.08.004

[35] Wang F, Zhang Z, Ma X, Zhang Y, Li X, Zhang X. Paths to open government data reuse: a three-dimensional framework of information need, data and government preparation. Inf Manag. 2023;60(8):103879. doi: 10.1016/j.im.2023.103879

[36] Wirtz BW, Weyerer JC, Becker M, Müller WM. Open government data: A systematic literature review of empirical research. Electron Mark. 2022;32(4):2381–404. doi: 10.1007/s12525-022-00582-8 36158525 PMC9487844

[37] Xu S, Liu J, Li S, Zheng Y. Barriers to and mechanism for open government data use by the private sectors: A grounded theory approach. Government Information Quarterly. 2025;42(3):102044. doi: 10.1016/j.giq.2025.102044

[38] Autant-Bernard C, Billand P, Frachisse D, Massard N. Social distance versus spatial distance in R&D cooperation: empirical evidence from European collaboration choices in micro and nanotechnologies. Papers in Regional Science. 2007;86(3):495–519.

[39] Powell WW, Koput KW, Smith-Doerr L. Interorganizational collaboration and the locus of innovation: networks of learning in biotechnology. Adm Sci Q. 1996;41(1):116–45.

[40] Owen-Smith J, Powell WW. Knowledge networks as channels and conduits: the effects of spillovers in the Boston biotechnology community. Organ Sci. 2004;15(1):5–21.

[41] Uzzi B. Social structure and competition in interfirm networks: the paradox of embeddedness. Adm Sci Q. 1997;42(1):35–67.

[42] Singh J. Collaborative networks as determinants of knowledge diffusion patterns. Manag Sci. 2005;51(5):756–70.

[43] Ter Wal ALJ, Boschma R. Applying social network analysis in economic geography: framing some key analytic issues. Ann Reg Sci. 2009;43(3):739–56.

[44] Fleming L, King CIII, Juda AI. Small worlds and regional innovation. Organ Sci. 2007;18(6):938–54.

[45] Sorenson O, Rivkin JW, Fleming L. Complexity, networks and knowledge flow. Research Policy. 2006;35(7):994–1017. doi: 10.1016/j.respol.2006.05.002

[46] Podolny JM. Networks as the pipes and prisms of the market. Am J Sociol. 2001;107(1):33–60.

[47] Granovetter MS. The strength of weak ties. Am J Sociol. 1973;78(6):1360–80.

[48] Burt RS. Structural holes and good ideas. Am J Sociol. 2004;110(2):349–99.

[49] Akerlof GA. The Market for “Lemons”: Quality Uncertainty and the Market Mechanism. The Quarterly Journal of Economics. 1970;84(3):488. doi: 10.2307/1879431

[50] Spence M. Job market signalling. Q J Econ. 1973;87(3):355–74.

[51] Digital and Mobile Governance Laboratory FU. China local public data openness and utilization report—cities. Shanghai: Fudan University. 2024. https://ifopendata.fudan.edu.cn/report

[52] Beijing Municipal Government. Beijing International Big Data Exchange officially established. 2021. https://english.beijing.gov.cn/investinginbeijing/two_zones/updates/202104/t20210413_2353482.html Accessed 2026 July 21.

[53] Shanghai Municipal People’s Government. National firsts for Shanghai’s newly opened data exchange. 2021. https://service.shanghai.gov.cn/sheninfo/specialdetail.aspx?Id=7929eda9-9692-4a19-93cd-9e865fd1d4ea Accessed 2026 July 21.

[54] National Development and Reform Commission of China, National Data Administration. Public Data Resource Authorized Operation Implementation Specification (Trial). 2025. https://www.ndrc.gov.cn/xxgk/zcfb/ghxwj/202501/t20250116_1395726.html

[55] Natural Earth. Free vector and raster map data. https://www.naturalearthdata.com/ Accessed 2026 June 27.

[56] Fan G, Wang XL, Zhu HP. NERI Index of Marketization of China’s Provinces: 2006 Report. Beijing: Economic Science Press. 2007.

[57] Goodman-Bacon A. Difference-in-differences with variation in treatment timing. J Econom. 2021;225(2):254–77.

[58] Butts K, Gardner J. did2s: two-stage difference-in-differences. R J. 2022;14(3):162–73. doi: 10.32614/RJ-2022-048

[59] Angrist JD, Pischke JS. Mostly harmless econometrics: an empiricist’s companion. Princeton: Princeton University Press. 2009.

[60] Khanna T, Palepu KG. Why focused strategies may be wrong for emerging markets. Harvard Business Review. 1997;75(4):41–51.

[61] Li Y, Yang Q, Wang C, Sun Y. Collaborative measurement of data opening policy in China’s municipal government data management system: Taking a regional central city as an example. PLoS One. 2023;18(8):e0289550. doi: 10.1371/journal.pone.0289550 37535651 PMC10399736

[62] Kawashita I, Baptista AA, Soares D, Andrade M. Open government data use: The Brazilian states and federal district cases. PLoS One. 2024;19(3):e0298157. doi: 10.1371/journal.pone.0298157 38442119 PMC10914293

[63] Zhang J, Li Y, Liu N, You J. Does digital government promote collaborative innovation? Evidence from the e-government pilot policy in China. PLoS One. 2025;20(10):e0334131. doi: 10.1371/journal.pone.0334131 41082538 PMC12517533

[64] Shen C, Yang Z, Lam F-I, Tam SW, Yang W, Li Y. Scientific and technological innovation cooperation network of the Greater Bay Area in South China: A social network analysis. PLoS One. 2025;20(7):e0326515. doi: 10.1371/journal.pone.0326515 40591554 PMC12212550

[65] Cohen WM, Levinthal DA. Absorptive capacity: a new perspective on learning and innovation. Adm Sci Q. 1990;35(1):128–52. doi: 10.2307/2393553

