# Supplementary material for: Who gets connected? Open government data and access to intercity innovation networks
Source: PLoS One. 2026 Sep 16;21(9):e0352359. doi: 10.1371/journal.pone.0352359 (PMC13580989; doi:10.1371/journal.pone.0352359)
Supplement: Supplementary file 1 [file pone.0352359.s003.docx]

**S1 Table. Sample construction and moderator-specific analytic samples**

| **Analysis sample** | **Cities** | **Observations** | **Inclusion requirement or scope** |
| --- | --- | --- | --- |
| Positive-collaboration outcome-source panel | 337 | 4,566 | City-years appear when the dyadic source records at least one intercity collaboration. |
| Zero-completed balanced outcome panel | 337 | 4,718 | Complete 337-city-by-2010–2023 skeleton; absent dyadic records are coded as zero. |
| Available economic-control sample | 298 | 4,085 | Requires GDP, population, tertiary-industry share, and urbanization. |
| Common sample used to compare covariate adjustment | 298 | 3,799 | Restricted to the full-control sample so columns differ only in included covariates. |
| Full-control baseline sample | 298 | 3,799 | Requires all eight time-varying city controls. |
| 2011 baseline-network moderator sample | 281 | 3,632 | Requires a 2011 partner-count record and all baseline controls. |
| 2011 commercial-credit moderator sample | 284 | 3,664 | Requires a 2011 CEI value and all baseline controls. |
| Financial-development moderator | 280 | 3,629 | Requires the 2011 financial-development moderator and all baseline controls. |
| Digital-economy moderator | 275 | 3,569 | Requires the 2011 digital-economy moderator and all baseline controls. |
| Market-support moderator | 281 | 3,632 | Requires the 2011 market-support moderator and all baseline controls. |
| Technology-contract moderator | 281 | 3,632 | Requires the 2011 technology-contract moderator and all baseline controls. |
| Highway-length moderator | 281 | 3,632 | Requires the 2011 highway-length moderator and all baseline controls. |

**Note:** The positive-source panel includes city-years with at least one recorded dyadic collaboration. The zero-completed panel adds absent city-years as zeros. Moderator samples require the relevant 2011 baseline measure and the full control set.
